# An economic evaluation of a hospital-wide bundle intervention to reduce hospital-acquired infections and bladder distension among hip fracture patients in Sweden

**DOI:** 10.1186/s13756-025-01573-y

**Published:** 2025-07-03

**Authors:** Sneha Abdul Jabbar, Maria Frödin, Ewa Wikström, Brigid M. Gillespie, Hanna Gyllensten, Annette Erichsen

**Affiliations:** 1 Sahlgrenska Academy, University of Gothenburg, Box 457, Gothenburg, SE-405 30 Sweden; 2 Institute of Health and Care Sciences, University of Gothenburg, Box 457, Gothenburg, SE-405 30 Sweden; 3 Department of Anesthesiology and Intensive Care Medicine, Sahlgrenska University Hospital, Västra Götalandregionen, Gothenburg, Sweden; 4 School of Business, Economics and Law, Department of Business Administration, University of Gothenburg, Västra Götalandregionen, Gothenburg, Sweden; 5 National Health and Medical Research Council Centre of Research Excellence in Wiser Wound Care, Queensland Gold Coast, Australia; 6 School of Nursing & Midwifery, Griffith University, Gold Coast, Australia; 7 Gold Coast University Hospital, Gold Coast Health Queensland, Gold Coast, Australia; 8 Sahlgrenska Academy, University of Gothenburg Centre for Person-Centred Care (GPCC), University of Gothenburg, Gothenburg, Sweden; 9 Department of Orthopeadic Research, Sahlgrenska University Hospital, Gothenburg, Sweden; 10 Institute of Health and Care Sciences, Sahlgrenska Academy, University of Gothenburg, Box 457, Gothenburg, SE-405 30 Sweden

**Keywords:** Bundle interventions, Hospital-acquired infections, Adverse events, Bladder distension, Catheter-associated urinary tract infections, Implementation costs, Cost-effectiveness analysis

## Abstract

**Background:**

A theory-driven knowledge translation program was established to co-create and implement evidence-based practices to prevent urinary catheter-associated urinary tract infections (UC-UTIs) and bladder distension (BD). This study investigates the cost-effectiveness of implementing the Safe Hands and Safe Bladder bundle intervention compared to standard care for patients undergoing hip fracture surgery in Sweden.

**Method:**

The study included outcomes from a quality register of patients who underwent hip fracture surgery at a Swedish hospital from 2015 to 2020. Adopting a healthcare perspective, estimates for the implementation cost were derived using activity-based costing, while the bundle’s cost-effectiveness was estimated using a decision tree model. Health outcomes were evaluated based on adverse events, specifically UC-UTI and BD. Analyses included calculating the incremental cost-effectiveness ratio (ICER), which denotes the incremental cost per added infection rate expressed as a percentage. Additionally, sensitivity analyses were conducted to test the robustness of the results under alternative cost assumptions.

**Results:**

The likelihood of avoiding BD or UC-UTI increased from 50 to 87% over the course of the intervention year. The discounted implementation cost was SEK 890,389 (corresponding to Int$ 102,721). However, the implementation cost was offset by costs for a prolonged hospital stay due to these adverse events, resulting in an overall cost savings of SEK − 7,334 per patient (Int$ -846) in 2020 compared to before the intervention was introduced. Consequently, the intervention proved to be cost-effective, leading to savings and a decrease in the occurrence of adverse events.

**Conclusion:**

Implementing the bundle intervention in units providing care for patients with acute hip fractures proved cost-effective. This offers decision makers valuable insights and demonstrates that implementation programs incorporating collaboration, facilitation and co-creation processes can effectively use limited resources. Further research should determine the generalizability of the findings to other settings and populations.

**ClinicalTrials.gov registration:**

NCT02983136 and ISRCTN 17,022,695, retrospectively registered after data collection were completed.

**Supplementary Information:**

The online version contains supplementary material available at 10.1186/s13756-025-01573-y.

## Highlights

This study analyzes the cost-effectiveness of a bundle intervention to prevent urinary catheter-associated infections and bladder distension in hip fracture surgery patients in Sweden, incorporating both implementation and intervention costs to offer a comprehensive evaluation of the economic impact.

The study demonstrates the value of collaboration, facilitation, and co-creation in implementing evidence-based practices in healthcare, highlighting potential cost savings and improved patient outcomes.

This study uses activity-based costing and decision tree modeling to evaluate the cost-effectiveness of implementation programs, providing a valuable methodology and insights into the economic feasibility and practical application of bundle interventions in healthcare.

## Background

Hospital-acquired adverse events are a significant challenge, threatening worldwide patient safety and healthcare systems [1]. Roughly five million hospital-acquired infections (HAIs) are projected to occur annually in acute care hospitals within Europe. This results in an additional 25 million days of hospitalization, leading to an economic burden ranging from EUR 13–24 billion [2]. According to statistical data, 10% of preventable patient harm in healthcare settings can be traced back to surgical procedures, with the majority of adverse events occurring before and after surgery [1]. Adverse events exacerbate patient suffering, morbidity, and mortality and are linked to prolonged hospitalization and elevated healthcare expenses. Patient safety strategies aim to reduce avoidable harm by fostering risk-awareness cultures and implementing safety measures [3]. Given the projected increase in the older people, who are particularly vulnerable to adverse events, the importance of improved adherence to these patient safety strategies becomes apparent [4, 5].

Urinary catheter-associated urinary tract infections (UC-UTIs) and bladder distension (BD) are prevalent and closely linked adverse events in healthcare settings, which can be effectively prevented through strict adherence to preventive measures by healthcare practitioners [6, 7]. The economic burden from UTIs significantly strains the healthcare system due to additional treatment costs and extended hospital stays. Approximately 66% of injuries recorded in adult inpatient care were characterized as preventable and associated with nursing-related factors. UTIs and BD constituted specific injuries within this nursing category, accounting for 12% and 6%, respectively [8]. Half of these cases require prolonged care, leading to an average increase of 6.1 to 12.0 days in hospital stays. This extended stay results in an additional 103,000 bed days and an annual cost of approximately SEK 600 million [8]. BD has proven to be a commonly observed event, particularly in orthopedic patients in the Swedish point prevalence measurements [9, 10]. This event might be caused by overly strict avoidance of indwelling urinary catheters to prevent UTIs [10].

Two overlapping theory-driven interventions, Safe Hands [11] and Safe Bladder [12, 13], were implemented in two steps at a university hospital in southwest Sweden. Their primary goal was to increase healthcare professionals’ adherence to measures to prevent UC-UTIs and BD. The interventions were associated with a reduced incidence of UC-UTIs from 18 to 4% over four years in patients undergoing surgery for a hip fracture [13]. The intervention also reduced BD from 41 to 9% within five years in the same population [12]. We have published two process evaluation papers [12, 13] and one manuscript [14], guided by the Medical Research Council’s guidance for process evaluation [15, 16], describing mechanisms of impact and contextual factors affecting the intervention. The present paper outlines the cost-effectiveness analysis of these interventions, which constitutes the final paper in this series.

Knowing the estimated cost data of implementation strategies and intervention-related expenses is vital for payers, policymakers, and providers, as it facilitates efficient resource allocation. Cost-effectiveness analysis plays a crucial role in determining the economic feasibility of healthcare interventions [17]. Numerous studies have highlighted the importance of assessing implementation costs. However, there is a lack of research on measuring costs from an implementation perspective [18–20].

Implementation costs are often overlooked in studies, as they are typically offset by the years of service delivery [21]. However, failure to account for these costs implies that the additional time required to implement this approach is considered insignificant and can be overlooked [18]. Neglecting implementation costs in economic evaluations can lead to higher costs per person and impact the cost-effectiveness of the intervention [21]. For effective resource allocation, decision makers should assess whether the intervention’s potential benefits justify the associated implementation and sustainability costs [22]. Incorporating the costs of implementation phases into the analysis is essential for ensuring a comprehensive assessment [23]. Therefore, this study aims to investigate the cost-effectiveness of implementing the Safe Hands and Safe Bladder bundle intervention compared to standard care for patients undergoing hip fracture surgery in Sweden. This investigation will explore implementation costs and healthcare use related to UC-UTIs and BD rates.

## Methods

The study employed a cost-effectiveness analysis of Safe Hands (ClinicalTrials.gov registration NCT02983136, registered 6 December 2016) and Safe Bladder (ISRCTN 17022695 registered 23 December 2023) interventions. Data were based on the secondary collected registry data from the two interventions conducted over five years (2015–2020). This study employed a before-after study design to evaluate the cost-effectiveness of the bundle intervention. Patients in the first year of the intervention, corresponding to 2015, before the adoption and adaptation of the intervention, are hereafter referred to as the standard care group. In contrast, those patients treated in 2020, following the implementation of the intervention, are hereafter referred to as the intervention group. This categorization allows for a comparative analysis between the standard care group and the intervention group, enabling a rigorous examination of the effectiveness and impact of the implemented intervention on patient outcomes. The methods and clinical results have been reported elsewhere [13, 24]. The design and reporting of the economic evaluation follow the Consolidated Health Economic Evaluation Reporting Standards (CHEERS) [25].

### Study setting and participants

The intervention setting was a university hospital specializing in orthopedic surgery in the southwest region of Sweden. This hospital performs approximately 10,000 orthopedic surgeries yearly, including 800–900 surgeries involving patients with acute hip fractures. The participating healthcare professionals worked in the units caring for patients with hip fractures, i.e., the operation department, emergency room, three ortho-geriatric wards and post-anesthesia care units/intensive care units. The interventions involved various participants, including department heads, managers, quality and safety coordinators, registered nurses and specialist nurses, anesthetists, orthopedic surgeons, and nurse assistants.

### The intervention and standard care

The two interventions were supported by theories on organizational culture, leadership, and dialogue and aimed to change healthcare professionals’ ways of thinking and acting regarding the prevention of UC-UTI and BD [26, 27]. An integrated knowledge translation approach was used as a strategy, involving collaboration and partnership between researchers and participants [28]. This collaboration resulted in the co-creation of two innovations in-line with recommended strategies [6, 7, 29, 30]: (i) a UC certificate on the electronic learning platform to prevent UTIs, and (ii) a nurse-driven UC protocol to ensure timely and appropriate insertion of an indwelling urinary catheter (IUC) using predefined IUC indications and a timely bladder scanning schedule to prevent BD. In the standard care approach, evidence-based practice for UC insertion was ensured by following national recommendations [12, 13]. Swedish regulations ensured the use of clean techniques, hand disinfection, and non-sterile gloves for UC insertion. Intermittent catheterization was the preferred method for urinary retention, while IUC was only permitted in a physician’s order [14].

### Data collection and management

All patients ≥ 65 years of age, admitted to ortho-geriatric wards for an acute hip fracture and cared for in the ortho-geriatric wards were included in the quality registry. Patients were selected based on specific inclusion and exclusion criteria (Table A.1).

The study used a secondary dataset of patients enrolled between 2015 and 2020 to estimate the health outcomes. For the cost-effectiveness analysis, patients in both the standard care and intervention groups were categorized into subgroups depending on the presence or absence of UTI, BD, both UTI and BD and none. To be classified as a member of the UC-UTI group, a physician must have diagnosed any UTI, the UTI occurred at least three days after admission, and the UTI had been associated with catheterization. The definition of BD in this study was consistent with the Swedish national trigger tool [31], which defines bladder distension as a urine volume of at least 500 ml twice or at least 1000 ml once or be diagnosed by a physician in cases where the patient had an IUC present at the time of discharge.

In this study, the costs associated with implementing the bundle intervention were identified using activity-based costing methods based on time used and participants involved in different activities conducted in the implementation process. The activities in the implementation process included leadership workshops, facilitating learning labs, co-creating the innovations and producing instructive films on UC, facilitating simulation scenarios and conducting skill tests, implementing UC certificates, and introducing nurse-driven protocols. The approximate salary levels of each type of participant were used to calculate the cost, including 50% social insurance and overhead costs. To account for the time value of money over a five-year period, the implementation costs were discounted at an annual rate of 3% [32].

The healthcare costs associated with inpatient admission were calculated according to Diagnosis Related Group (DRG) weights, using the NordDRG [33] lists to determine the code corresponding to hip fracture in 2020. The appropriate DRG code for hip fracture was identified as H02C, with a corresponding weight of 1.7624 in 2020. The cost per unit of weight was further determined to be SEK 63,634. This weight was multiplied by a predetermined “cost per 1 DRG” to determine the hip fracture treatment cost [34, 35]. In this study, we opted to use the cost for the year 2020 for both the intervention and standard care groups to minimize the potential impact of between-year differences caused by, e.g., inflation, DRG coding, or changes in treatment pathways that were unrelated to the implementation program.

To evaluate the additional treatment costs incurred by patients due UTI and BD, the additional length of hospital stays and the cost per day for patients admitted to the orthopedic department were considered. Published literature was searched strategically to identify studies that investigated the impact of hospital-acquired UTI and BD on the hospital length of stay (HLOS), as well as studies that could serve as benchmarks for cost-effectiveness analysis. Thus, HLOS due to UTI and BD was assumed to increase by 3.0 days and 1.6 days, respectively, based on the findings of the literature review [36, 37]. The cost per day was obtained from the price lists for services as per the inter-regional cooperation agreement on healthcare within Västra Sjukvårdsregionen [38]. The daily treatment cost in 2022 was adjusted to the 2020 value using an inflation factor of 0.92 [39] to ensure a common value year. This led to a daily cost of SEK 7946 for the orthopedic department [38]. The bundle intervention’s cost-effectiveness relative to standard care was evaluated by adopting a healthcare perspective, given that each patient was only followed for a short period, and no discounting was given for treatment costs.

The clinical experience of the research group indicated that approximately 70% of the 10,000 yearly orthopedic department admissions involved intermittent or indwelling catheterization. Thus, to put the results in perspective to the annual patient population, treatment costs for 7,000 patients were calculated for years one and five of the intervention.

### Data analysis

**Fig. 1:**
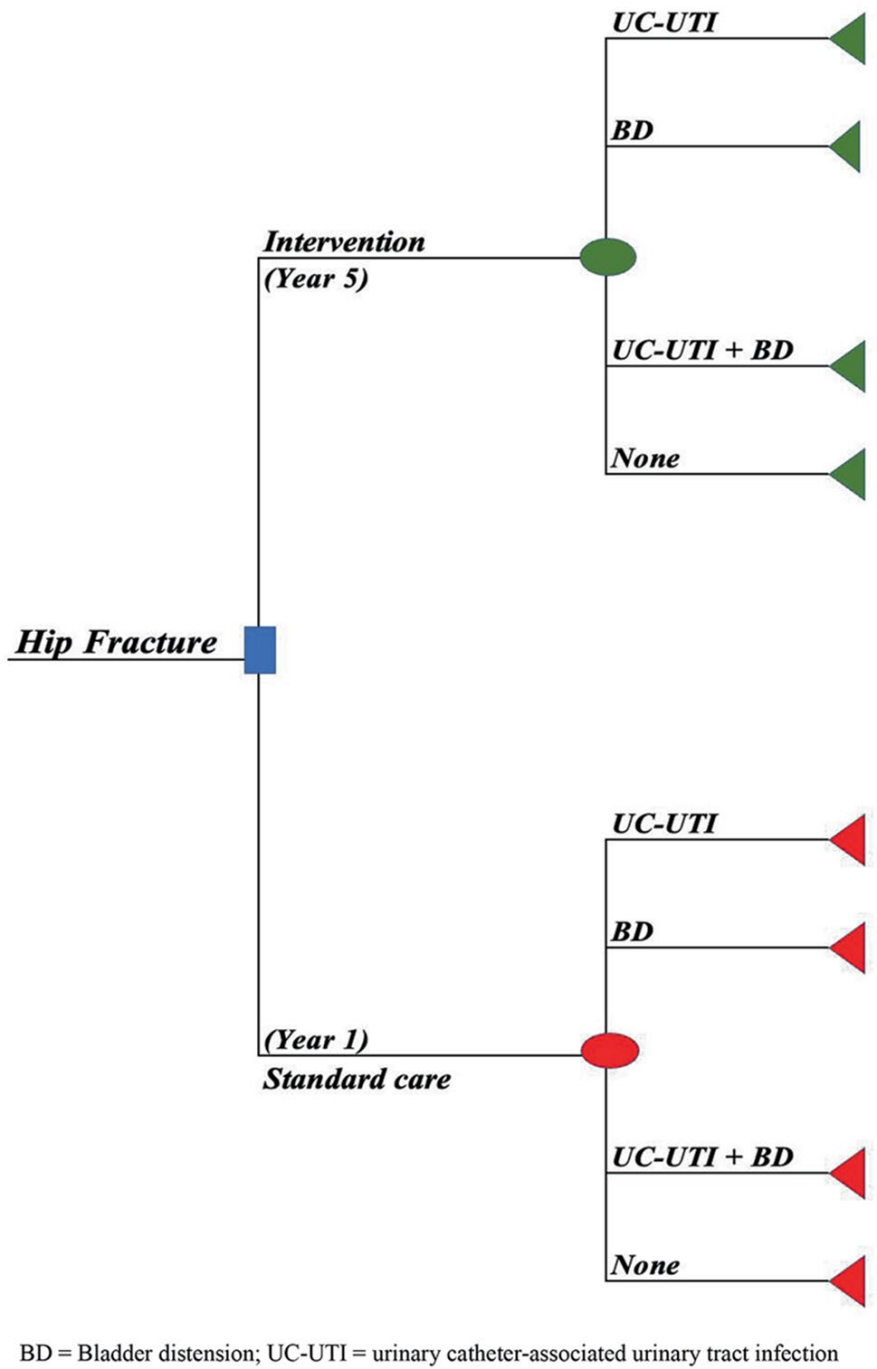
Decision Tree Model BD = Bladder distension; UC-UTI = urinary catheter-associated urinary tract infection

The bundle intervention’s cost-effectiveness relative to standard care was evaluated using a decision analytical model, i.e., a decision tree model (Fig. 1). The decision tree model starts with hypothetical hip fracture patients being recruited from the data set and entering the model upon diagnosis. Patients from the first year of the bundle intervention go into the model according to the standard care branch, whereas patients from year five follow the intervention branch. The decision tree’s transition probabilities are based on hospital hip fracture data. These probabilities were determined by computing the likelihood of mutually exclusive outcomes (such as developing UTI, BD, both, or none) within the intervention and standard care groups.

The cost of treatment for each potential outcome is calculated and added to each branch. The decision tree was used to calculate the cost-effectiveness of the intervention as an incremental cost-effectiveness ratio (ICER) in which the difference in costs is divided by the difference in health outcomes. In this case, ICER was interpreted as an additional cost per percentage reduction in infection. Because there is no willingness-to-pay threshold for reducing HAI in Sweden, the ICER was compared against a range of different willingness-to-pay thresholds from SEK 0 to SEK 100,000. The results are presented in SEK and international Dollars. The conversion to international dollars was conducted using the Purchasing Power Parity (PPP) of Sweden for the year 2020, which was estimated at 8.436 (i.e., SEK 8.436 being equivalent to one international dollar) [40].

To perform a sensitivity analysis of assumptions made during the analysis, a one-way deterministic sensitivity analysis was conducted by changing one input parameter at a time by +/- 20% while keeping all other input parameters as base case values to evaluate the impact of uncertainty on the ICER. Additionally, a probabilistic sensitivity analysis (PSA) involving 1000 simulations was conducted to examine the effects on the ICER when underlying variables simultaneously vary across plausible ranges and distributions.

## Results

Some 2166 work hours were reported during the five-year implementation period. The total cost of implementing Safe Hand’s intervention amounted to SEK 941,082 (Discounted SEK 890,389; Int$ 105,546), assuming 50% social insurance and overhead costs (Table 1).

**Table 1:** Five-Year implementation overview: participants, activity hours, and total costs in SEK

Timeline	Year	Activities	Participants	Number of Participants	Activity Hours	Unit Cost (SEK)	Cost (SEK)	Discounted Costs (SEK)
Mid 2015–2016	1	Workshop-Leadership	Operations Head	3	48	1194	28 644	
		8-week observation	Unit Chief	7	112	708	66 240	
			Lab Leader	1	16	364	8 733	
			Research and execution staffs	4	298	974	137 691	
			**Subtotal**	**15**			**241 309**	**241 309**
2016–2017	2	Facilitating 11 learning labs	Research and execution staffs	15	145	928	51 693	
		Co-creating SOP	Manager	2	14	259	5 449	
		Safe bladder initiated	Medical experts	8	60	1389	32 448	
			**Subtotal**	**25**			**89 590**	**86 980**
2016–2017	3	E-learning for UC	Research and execution staffs	25	244	921	76 462	
		Learning materials	Management	4	2	337	1 012	
		Instructive film	Photographer	1			80 000	
		Skill tests, simulations						
		10 leaning labs						
			**Subtotal**	**30**			**157 474**	**148 435**
2 018	4	Implementing UC certificate	Research and execution staffs	974	1201	1479	369 163	
		Simulation tests	Material cost				60 000	
		Nurse- driven protocol introduced						
		Timely bladder scanning						
			**Subtotal**	**974**			**429 163**	**392 745**
2019–2020	5	Feedback and post-measurement	Research and execution staffs	48	24	195	7 005	
		Handing over intervention	Instructor	1	50	221	16 541	
			**Subtotal**	**49**			**23 546**	**20 920**
			**Total**				**941 082**	**890 389**

In year one of the intervention, 406 eligible patients were enrolled in the study, while in year five, the number increased to 626 eligible patients. The probability of not developing adverse events increased from 50% in year one to 87.4% in year five, resulting in an incremental decrease of 38% (Table 2).

**Table 2:** Disease probabilities comparison: year 1 vs. year 5

Disease Category	Count in Year 1, *n* = 406	Probability in Year 1	Count in Year 5, *n* = 626	Probability in Year 5	Incremental Effect
UTI	36	0,089	22	0,035	-0,054
BD	126	0,310	54	0,086	-0,224
UTI + BD	39	0,096	3	0,005	-0,091
None	201	0,495	547	0,874	0,379

After assigning costs and probabilities of health outcomes to the decision tree, the folded back cost for year one (before) was calculated to be SEK 121,719 (equals Int$ 14,429), and for year five (after), it was SEK114,258 (Int$ 13,544) (Table A.2 and Table A.3), leading to an incremental cost of SEK − 7334 (Int$ -869). The corresponding cost for 7,000 patients during years one and five was SEK 852,033,272 and SEK 800,697,770, respectively (Table 3).

**Table 3:** Disease probabilities and costs for year 1 and year 5

Category	Standard Care (Year 1)	Intervention (Year 5)
Costs (SEK)		
Total expected cost	121 719 SEK	114 258 SEK
Cost for 7000 people	852 033 271 SEK	799 807 380 SEK
Intervention and implementation cost		800 697 769 SEK
Cost per person	121 719 SEK	114 385 SEK
Disease Probabilities		
Total diseases	0,50	0,13
No Infection	0,50	0,87
All values are rounded		

**Fig. 2:**
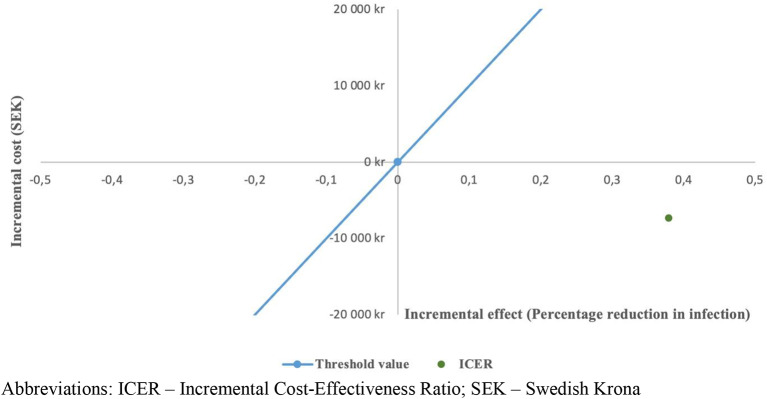
Cost-effectiveness plane with the line indicating willingness-to-pay threshold of SEK ± 100,000 per percentage reduction in rate of infection. Abbreviations: ICER – Incremental Cost-Effectiveness Ratio; SEK – Swedish Krona

The combined analysis of costs and health outcomes resulted in an ICER of SEK − 19,364 (Int$ -2,295) per avoided healthcare-associated infection. Thus, the intervention was cost-saving while also reducing the incidence of infections. By employing the cost-effectiveness plane as a visual aid, the intervention outcome is situated in the southeast quadrant (refer to Fig. 2), a scenario frequently characterized as dominating the control condition.

**Fig. 3:**
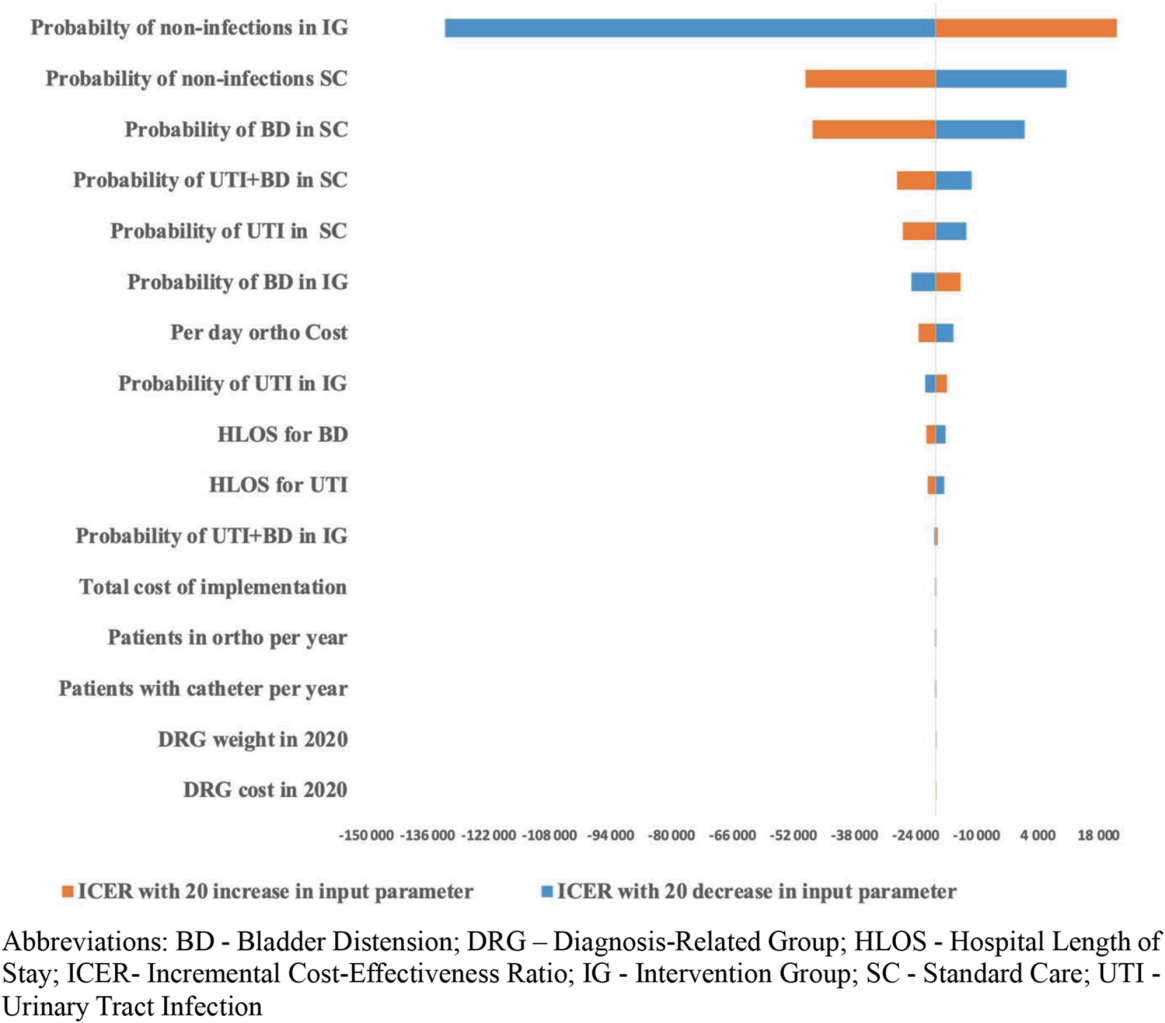
Tornado Diagram showing results from the Sensitivity Analysis. Abbreviations: BD - Bladder Distension; DRG – Diagnosis-Related Group; HLOS - Hospital Length of Stay; ICER- Incremental Cost-Effectiveness Ratio; IG - Intervention Group; SC - Standard Care; UTI - Urinary Tract Infection

Modifying the input parameter for the probability of non-infection in the intervention group by a 20% increment (equivalent to a 20% decrease in the intervention’s effectiveness) led to an ICER of SEK 22,160. In comparison, a 20% decrement in the parameter resulted in an ICER of SEK − 132,044. Similarly, modifying the parameter probability of non-infection in standard care by an increase of 20% resulted in an ICER of SEK − 49,268. In comparison, a 20% decrement in the parameter resulted in an ICER of SEK 10,540. Changing the parameter probability of BD in standard care by an increase of 20% resulted in an ICER of SEK − 47,634, and the decrease in the parameter by 20% resulted in an ICER of SEK 945. The input parameters with the least impact on the ICER were DRG weight and cost, the total cost of implementation, and patients with catheters per year (Fig. 3). The direction of these ICERs is primarily influenced by the hypothetical threshold used for comparison. Because there is no specific willingness-to-pay threshold for reducing healthcare-associated infections in Sweden, the ICERs were compared against a series of hypothetical thresholds.

**Fig. 4:**
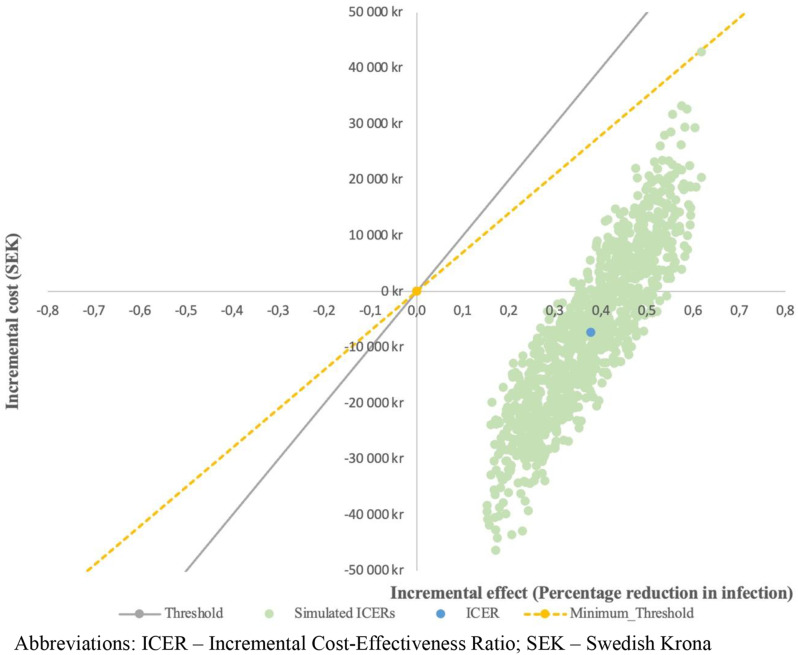
Cost-effectiveness plane showing results from probabilistic sensitivity analysis. Abbreviations: ICER – Incremental Cost-Effectiveness Ratio; SEK – Swedish Krona

Based on the results of the PSA, a portion of the simulated ICERs remained in the southeast quadrant of the cost-effectiveness plane (i.e., the intervention remains more effective and less costly than standard care). However, some ICERs were in the northeast quadrant, suggesting that the intervention was more effective but costly than the standard care group (Fig. 4). Based on the PSA findings, we can estimate that if the societal willingness-to-pay is at least SEK 70,000 per percentage decrease in the studied complications, this intervention will consistently be considered cost-effective in 100% of the simulations.

## Discussion

This study primarily reports two key findings. First, the introduction of bundle interventions incurred a significant cost of SEK 941,082 (Int$ 111,555) at the current 2020 value. This amount was determined by applying an activity-based costing method, which considered salaries, social insurance, and overhead expenses. Applying a standard discount rate of 3% to account for the time value of money, the implementation cost was reduced to SEK 890,389 (Int$ 105,546). Second, the cost-effectiveness analysis indicates that the interventions were cost-saving and more effective than standard care. Over time, they contributed to a reduction in UTIs and BD. The analysis revealed a decrease in infection probability from 50 to 12% and an increase in the likelihood of avoiding infection from 50 to 87%. The incremental cost amounted to SEK − 7333 (Int$ -849), and the intervention was deemed dominant, signifying it was more effective and less costly than standard care. Moreover, sensitivity analysis shows that despite notable cost fluctuations, the intervention would remain cost-effective if a threshold of at least SEK 70,000 is accepted.

This study demonstrates notable strengths, particularly in its integral role in enhancing patient safety, signifying a process internally driven within the health system rather than stemming from external interests. This intrinsic integration facilitated its direct implementation into routine care. A cost-effectiveness analysis, conducted from a healthcare perspective, also applies to healthcare management and decision makers. A quasi-experimental before/after design with historical controls was used because it was impractical to randomize participants to each study group. The initial implementation of the initiative in a smaller segment of the hospital, which later expanded to all units caring for patients with hipfracture, posed a constraint on the feasibility of integrating an external register-based comparator to address the observed reduction in HLOS.

This study incorporated supplementary data from another hospital region to overcome data limitations. Due to unavailable concurrent cost data, DRG costs from 2020 were used for intervention and standard care groups to ensure comparability. This study assumed all patients had surgery within the same year, enabling a controlled cost-effectiveness evaluation. It was also assumed that cost deviations from the mean were evenly distributed across both study populations. However, it is important to acknowledge that the DRG cost approach lacks specificity for individual patients, as it mainly estimates costs per episode for a cohort with comparable treatment. Consequently, the dynamic shifts in the patient demographic can present difficulties in accurately reflecting the true costs [40].

Moreover, the implementation costs were discounted to year one, whereas healthcare costs were calculated based on their 2020 value, potentially not fully capturing the study’s multi-year implementation. However, sensitivity analysis indicated that removing the discounting did not yield significantly different results. Further research is needed to determine the optimal discounting approach. Despite limitations, DRG costs have several healthcare advantages. The DRG offers a quantifiable service definition, accurate cost estimation, objective performance assessment, and transparent payment systems, enhancing accountability in healthcare financing [41]. The precision and validity of economic evaluation results depend on the quality of the inputs used. Thus, it is imperative to determine the suitability of incorporating DRG cost databases. Špacírová et al. (2022) suggest that unit costs for economic evaluations should ideally demonstrate transparency, methodological rigor, accurate reflection of opportunity costs, timely publication, and comprehensive reporting of uncertainty to meet standards [42]. The authors’ study relies on the widely accessible DRG costing system, which guarantees broad availability at both national and regional levels. The annual presentation of statistics enhances reliability and accessibility. Given these factors, adopting DRG-based costing stands out as a robust methodology for economic evaluations in the context of this study [42].

The study is further limited by its dependency on data from a solitary center, thus affecting the potential applicability of the findings. Moreover, the analyses were conducted using aggregated data based on a quality registry rather than on individual patient-level data. It was assumed that the characteristics of the groups remained fairly consistent across the years of study. The quality of the present study also relies on the rigor applied in evaluating the effectiveness of the intervention [12, 13]. It is important to note that these studies did not assess adherence to the implemented Standard Operating Procedures. Instead, effectiveness was measured by changes in the rates of UTI and BD.

No patient and public involvement specific to this project was included, as the research focused on implementation within standard care. Consequently, co-creation in partnership with staff was a core component of the implementation process [11], while patient representation was deemed relevant only within the existing standard care structures for patient involvement. Additionally, the absence of post-discharge follow-up and failure to consider long-term outcomes or costs beyond the intervention period, such as outpatient care expenses or productivity loss, could influence the overall assessment of the intervention’s cost-effectiveness. However, unless there are significant adverse events in the future, it is reasonable to expect these results to continue or possibly become even stronger over time. The lack of adverse events should not inherently contribute to higher resource consumption in future periods. The main conclusion drawn from this analysis is that the impact of implementation costs on overall patient expenditure is so minimal that they lean towards being negligible. This observation becomes more significant, especially when considering the long-term benefits for patients, notwithstanding the initial substantial appearance of implementation costs in the short term.

Furthermore, the decision tree used includes assumptions of the uniform distribution of changes between groups that may not accurately mirror the study environment. The single value year and DRG-based calculation were introduced to address known trends in healthcare resource use and costs. The anticipated impact of the absence of a temporal component in decision trees is likely to be mitigated within the examined population, given the short duration of the patient episodes under study. Thus, the decision tree allows for a systematic evaluation of the intervention’s cost-effectiveness using precise patient-level data. It accommodates variability and uncertainty in decision-making processes [43].

The sensitivity analysis provides a robustness check of the results, giving a range of outcomes with input parameters. The one-way sensitivity analysis identified three crucial parameters that substantially influenced the intervention’s cost-effectiveness. These parameters are the probability of non-infection occurring in both the intervention and standard care groups and the probability of BD in the standard care group. These findings imply that the fluctuations in these specific parameters can drive the cost-effectiveness metrics to levels that might raise concerns about implementing the intervention. The feasibility of the intervention may be compromised if the associated costs experience a substantial increase, which was observed in certain cases, causing ICERs to shift from negative to positive. Like the probabilistic sensitivity analysis, most simulations showed the intervention as dominant, but some indicated the intervention was more effective and costlier. Nevertheless, most ICERs remained lower than our assumed threshold for willingness to pay, thereby suggesting cost-effectiveness. Overall, the findings suggest investing in infection prevention and control can be a cost-saving strategy while improving patient outcomes.

This study is similar to research on interventions to improve hand hygiene and reduce healthcare-associated infections. However, it differs by focusing on a specific study population and targeted infections [44–46]. Studies by Anita Huis and Thi Anh aimed to improve hand hygiene in healthcare professionals and reduce HAIs without focusing on specific target populations [44, 46]. Ling conducted a study to decrease catheterization rates without specifically targeting infections [45]. The patient populations in these studies varied from inpatient hospital settings to academic hospitals. In contrast, our study targeted hip fracture patients to reduce catheter-associated UTIs and BD. As supported by previous research, we recognize the importance of incorporating preventive measures to mitigate both these adverse events [10]. Our study aligns with earlier research [44–46], as the interventions employed were consistent with effective strategies, improved patient outcomes, and generated long-term cost savings.

Moreover, Huis et al. (2013), this intervention resulted in a further decrease of 2.7% in the HAI rate. They reported a program cost of EUR 364,668 from the team- and leader-directed strategy and an ICER of EUR 2074 per percent decrease in HAIs. Similarly, the intervention implemented by Ling was also successful in reducing catheter associated UTI [45]. The program cost of this intervention was reported to be AUD 32,810 and an ICER of AUD 264 at nine months post-intervention. In comparison, our study significantly reduced hospital-acquired infections by 37%, with a negative ICER of SEK − 19,364, indicating that our intervention was more effective and less costly than standard care. This raises possibilities regarding the potential success of our intervention. One possible explanation for these positive outcomes could be the careful selection of the target population or the detailed implementation of the bundled intervention approach. These factors may have contributed to the observed favorable results. The enabling factors we identified were the integrated knowledge translation approach, facilitation, and creating safe spaces for collaborative learning [14].

Our study employed a similar approach to previous work in calculating interventions and hospital-associated infection costs. We considered program, and UTI and BD costs, which is consistent with the methodology used in previous studies. However, the cost components may vary across studies. Other studies [44–49] examined different cost elements, including personnel, training, materials, and specific interventions and procedures. In contrast, our analysis did not include expenses related to hand rub, saline, chlorhexidine, and catheters, which were factored into other studies. Nevertheless, an examination was conducted looking into the costs of hand hygiene materials during the initial phase. However, owing to their minimal expense and the challenge of evaluating their usage per patient, these expenses were omitted from our analysis. While a comprehensive assessment of the intervention’s total cost would require consideration of all potential cost components, including indirect and long-term expenses, our study was limited to direct healthcare costs associated with program implementation, focusing on the major cost components to the health system, including staff hours and hospitalization costs. However, it is important to acknowledge this limitation in our study and recognize that the cost estimates presented here do not entirely capture the total cost of implementing the intervention. The cost of implementing the intervention may vary depending on several factors, such as the specific context in which the intervention is being implemented and the resources available. Yet, the available evidence suggests that the intervention’s cost per patient benefiting from it is likely to be relatively low. Additionally, the intervention has the potential to generate significant cost savings in the long run by reducing the need for HLOS and other costly medical services. The intervention should thus be possible to implement without exceeding the healthcare budget and potentially even generate savings. Nonetheless, the successful execution would demand that decision makers allocate funding during the initial phase before witnessing the positive impacts; otherwise, there is a potential for delay or obstruction in the implementation process [18].

## Conclusion

Our findings suggest that the implementation interventions (Safe Hands and Safe Bladder) were less costly than standard care while reducing UC-UTIs and BD among patients undergoing hip fracture surgery. The intervention was cost-effective, with the potential benefits outweighing the implementation costs. Thus, the bundle intervention should be a valuable addition for decision makers considering interventions to reduce the incidence of HAIs. Subsequent investigation could explore the generalizability of these findings to other healthcare settings and diverse patient populations.

## Electronic supplementary material

Below is the link to the electronic supplementary material.

Supplementary Material 1

## Data Availability

No datasets were generated or analysed during the current study.

## Declarations

**Human Ethics and Consent to participate**.

The study approval was granted by the Ethical Review Board in Gothenburg, Sweden (reference No: 166 − 15 and 327 − 17, amendment No: 2022-0270-02). The four principal requirements of the Helsinki Declaration, which are autonomy, non-maleficence, beneficence, and justice, were upheld in this study [50]. Healthcare professionals were provided with written information stating their participation was voluntary and they could withdraw from the study anytime. Similarly, patient data were collected from a local quality registry, and patients were given written information about the use of their data and informed about the process for withdrawing their participation or opting out of data use. However, this project was conducted within the framework of improved patient safety, thereby limiting the feasibility of patients explicitly expressing a preference against adherence to bundle intervention by the healthcare professionals.
