# Supplementary material for: An economic evaluation of a hospital-wide bundle intervention to reduce hospital-acquired infections and bladder distension among hip fracture patients in Sweden
Source: Antimicrob Resist Infect Control. 2025 Jul 3;14:80. doi: 10.1186/s13756-025-01573-y (PMC12226840; doi:10.1186/s13756-025-01573-y)
Supplement: Supplementary file 1 — Supplementary Material 1 [file 13756_2025_1573_MOESM1_ESM.docx]

**An Economic Evaluation of a Hospital-wide Bundle Intervention to Reduce Hospital-Acquired Infections and Adverse Events among Hip Fracture Patients in Sweden**

Table of Contents

[Table A.1: Extracted Variable and Exclusion Criteria for Patient Inclusion in the Study. 1](#_Toc168831170)

[Table A.2: Probabilities and Expected CostS for the Year 2020 1](#_Toc168831171)

[Table A.3: Probabilities and Expected Costs for the Year 2016 2](#_Toc168831172)

## **Table A.1: Extracted Variable and Exclusion Criteria for Patient Inclusion in the Study.**

|  | **Safe Hands** | **Safe Bladder** |
| --- | --- | --- |
| **Extracted Variable** | Age (Years)  Sex (Male or Female)  ASA Classification score (1-4)  DM Type 1 and 2 (Yes or No)  Hospital length of stay (Days)  Type of catheterization Treatment (IUC, IC, or IUC + IC)  Catheter days (including catheterization)  Number of IC, UC - UTI | Risk of bladder distension (Yes or No)  Urine volume ≥500 ml (Yes or No)  Largest volume observed during hospital stay (ml)  Urine volume documented when inserting IUC (ml)  Documented catheter indications and removal plan  Catheter present on discharge |
| **Exclusion Criteria** | Surgery at another hospital or residing outside Sweden  Distal fracture  Resection arthroplasty or previously included due to contralateral fracture  Age less than 65  Intermittent self-catheterization preadmission  Chronic or suprapubic catheter  Urostomy or dialysis  No catheterization or catheter inserted at another ward or hospital  Hospital stays ≤2 days | |
| Abbreviations:  ASA – American of Anaesthesiologists physical status classification system; DM – Diabetes Mellitus; IC – Intermittent Catheterization; IUC – Indwelling Urinary Catheter; UC – Urinary Catheter, UTI – Urinary Tract Infection | | |

##

## **Table A.2: Probabilities and Expected Costs for the Year 2020**

| **Categories of Cases** | **Probability** | **Cost per person** | **Expected cost** |
| --- | --- | --- | --- |
| **UTI** | 0,035 | 135 987 SEK | 4 779 SEK |
| **BD** | 0,086 | 124 862 SEK | 10 771 SEK |
| **UTI and BD** | 0,005 | 148 700 SEK | 713 SEK |
| **No Infection** | 0,874 | 112 149 SEK | 97 996 SEK |
| **Total** | 1,000 |  | 114 258 SEK |
| Abbreviations:  BD – Bladder Distension; SEK – Swedish Kronor; UTI – Urinary Tract Infection | | | |

## **Table A.3: Probabilities and Expected Costs for the Year 2016**

| **Categories of Cases** | **Probability** | **Cost per Person** | **Expected Cost** |
| --- | --- | --- | --- |
| **UTI** | 0,089 | 135 987 SEK | 12 058 SEK |
| **BD** | 0,310 | 124 862 SEK | 38 750 SEK |
| **UTI and BD** | 0,096 | 148 700 SEK | 14 284 SEK |
| **No Infection** | 0,505 | 112 149 SEK | 56 627 SEK |
| **Total** | 1,000 |  | 121 719 SEK |
| Abbreviations:  BD – Bladder Distension; SEK – Swedish Kronor; UTI – Urinary Tract Infection | | | |
